# A modified M-stage classification based on the metastatic patterns of pancreatic neuroendocrine neoplasms: a population-based study

**DOI:** 10.1186/s12902-018-0301-z

**Published:** 2018-10-19

**Authors:** Xianbin Zhang, Jiaxin Song, Peng Liu, Mohammad Abdul Mazid, Lili Lu, Yuru Shang, Yushan Wei, Peng Gong, Li Ma

**Affiliations:** 1 The First Affiliated Hospital of Dalian Medical University, Zhongshan 222, Dalian, 116011 China; 2 Institute for Experimental Surgery, Rostock University Medical Center, Schillingallee 69a, 18057 Rostock, Germany; 3 Department of Epidemiology, Dalian Medical University, Lvshun West 9, Dalian, 116044 China; 4 Department of Evidence-based Medicine and Statistics, the First Affiliated Hospital of Dalian Medical University, Zhongshan 222, Dalian, 116011 China; 5 Department of General Surgery, the Shenzhen University General Hospital and Shenzhen University School of Medicine, Xueyuan 1098, Shenzhen, 518055 China; 6 Department of Epidemiology, Dalian Medical University, Zhongshan Road 222, Dalian, 116011 China

**Keywords:** Metastasis, Survival, Prognosis, Pancreas, Cancer

## Abstract

**Background:**

The present study aims to improve the M-stage classification of pancreatic neuroendocrine neoplasms (pNENs).

**Methods:**

Two thousand six hundred sixty six pNENs were extracted from the Surveillance, Epidemiology, and End Results database to explore the metastatic patterns of pNENs. Metastatic patterns were categorized as single, two, or multiple (three or more) distant organ metastasis. The mean overall survival and hazard rate of different metastatic patterns were calculated by Kaplan-Meier and Cox proportional hazards models, respectively. The discriminatory capability of the modified M-stage classification was evaluated by Harrell’s concordance index.

**Results:**

The overall survival time significantly decreased with an increasing number of metastatic organs. In addition, pNENs with only liver metastasis had better prognosis when compared to other metastatic patterns. Thus, we modified the M-stage classification (mM-stage) as follows: mM_0_-stage, tumor without metastasis; mM_1_-stage, tumor only metastasized to liver; mM_2_-stage, tumor metastasized to other single distant organ (lung, bone, or brain) or two distant organs; mM_3_-stage, tumor metastasized to three or more distant organs. Harrell’s concordance index showed that the modified M-stage classification had superior discriminatory capability than both the American Joint Committee on Cancer (AJCC) and the European Neuroendocrine Tumor Society (ENETS) M-stage classifications.

**Conclusions:**

The modified M-stage classification is superior to both AJCC and ENETS M-stage classifications in the prognosis of pNENs. In the future, individualized treatment and follow-up programs should be explored for patients with distinct metastatic patterns.

**Electronic supplementary material:**

The online version of this article (10.1186/s12902-018-0301-z) contains supplementary material, which is available to authorized users.

## Background

Pancreatic neuroendocrine neoplasms (pNENs) are relatively rare tumors. However, a recent population study showed that the incidence of pNENs increased more than 4-fold from 1973 to 2012 [1]. Moreover, pNENs are considered the most serious neuroendocrine neoplasms (NENs), due to the patients have a shorter median overall survival times (3.6 years) when compared to those with tumors located in lung (5.5 years), rectum (24.6 years), and appendix (more than 30.0 years) [1].

Cancer staging classification systems are used to codify the extent of cancer. They allow clinicians to quantify prognosis and plan treatment for individual patients. Two widely used tumor staging classification systems, which are proposed by the American Joint Committee on Cancer (AJCC) and the European Neuroendocrine Tumor Society (ENETS), describe M_0_-stage as having no distant metastasis and M_1_-stage as having at least one distant metastasis [2, 3]. However, several studies demonstrated that pNENs with liver metastasis have better prognosis than other metastatic patterns [4–6].

Therefore, we utilized the Surveillance, Epidemiology, and End Result (SEER) database to explore the prognosis of different metastatic patterns of pNENs and propose a modified M-stage classification. This modified M-stage classification proves to be superior to both AJCC and ENETS M-stage classifications in prognosis.

## Methods

### Study cohort

As published previously [3], we utilized the topography codes (C25.0 to C25.9) and histology codes (8150, 8151, 8152, 8153, 8154, 8155, 8156, 8157, 8240, 8241, 8242, 8243, 8244, 8245, 8246, and 8249) of the International Classification of Diseases for Oncology (third edition) to identify pNENs.

### Outcomes and variables

The primary outcome was overall survival. Demographic data included age, sex, and race; tumor characteristics included tumor size, primary site, differentiation, 7th AJCC T-stage, and N-stage; treatment information included surgery and radiotherapy. Single organ metastasis was defined as the tumor spreading from pancreas to another single distant organ [7]. Similarly, two organ metastases were defined as the tumor spreading from pancreas to two distant organs. Tumors spreading from pancreas to three or more distant organs were defined as multiple metastases.

### Inclusion and exclusion criteria

Patients microscopically diagnosed as pNENs were included in the present study. We excluded cases with unclear or incomplete information about metastasis. In addition, we also excluded cases without information about survival time.

### Statistical analyses

To compare the constituent ratio of variables among patients, we broke the continuous variables (age, tumor size) into binary variables. Survival time was plotted using the Kaplan-Meier estimator and Cox proportional hazards model. The results were presented as mean and hazard ratio, respectively, each with a 95% confidence interval (CI). Harrell’s concordance index was used to evaluate the discriminatory capability of the modified M stage classification. An index value of greater than 0.70 suggests the classification has an acceptable discriminatory capability [8]. Differences with *P* ≤ 0.05 divided by the number of meaningful comparisons, Bonferroni correction, were considered to be significant. Differences with *P* ≤ 0.1 divided by the number of meaningful comparisons, were considered to indicate a tendency. All statistical analyses were performed using SPSS 19.0 (IBM, New York, USA) or R (version 3.5.0).

## Results

### Patient characteristics

**Fig. 1:**
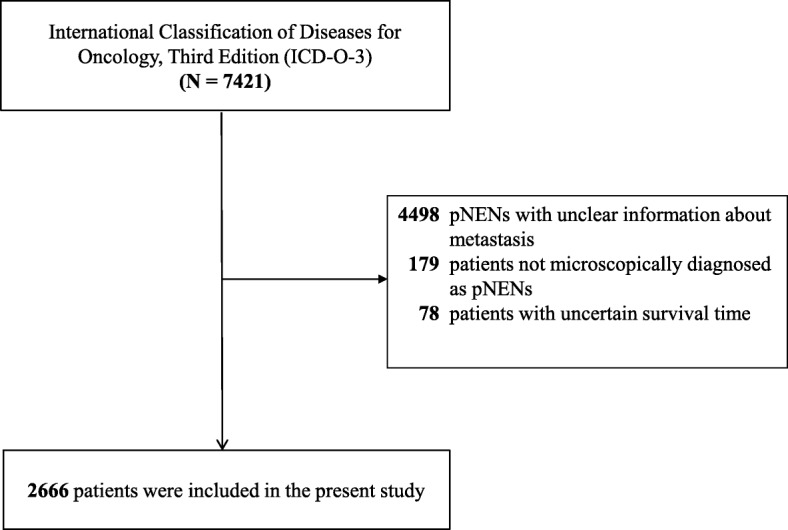
Flow chart of patient selection

In total, 2666 patients (mean age 60.9 years ±13.6 years; 55.7% male, 78.8% white) were included in the present study (Fig. 1). Many patients (55.4%) underwent surgery, and some (4.7%) were treated with radiation. The constituent ratios of tumor size, location, differentiation, T-stage, and N-stage were significantly (*P* < 0.05) different between patients with and without metastasis (Table 1).

**Table 1:** Clinicopathological Characters

	Without Metastasis	Metastasis	*P*
	*N* = 1679	*N* = 987	
Age (years)			0.221^b^
≤ 60	793 (47.2%)	442 (44.8%)	
> 60	886 (52.8%)	545 (55.2%)	
Sex			0.338^a^
Male	924 (55.0%)	562 (56.9%)	
Female	755 (45.0%)	425 (43.1%)	
Race			0.011^a^
White	1314 (78.3%)	787 (79.7%)	
Black	191 (11.4%)	130 (13.2%)	
Other	174 (10.3%)	70 (7.1%)	
Size (cm)			< 0.001^b^
≤ 2	670 (39.9%)	68 (6.9%)	
> 2	934 (55.6%)	699 (70.8%)	
Unclear	75 (4.5%)	220 (22.3%)	
Primary Site			< 0.001^b^
Head	502 (29.9%)	258 (26.2%)	
Body	295 (17.6%)	106 (10.7%)	
Tail	542 (32.3%)	314 (31.8%)	
Other	340 (20.2%)	309 (31.3%)	
Differentiation			< 0.001^b^
Well	1043 (62.1%)	189 (19.1%)	
Moderately	221 (13.2%)	86 (8.7%)	
Poorly	64 (3.8%)	95 (9.6%)	
Undifferentiated	17 (1.0%)	28 (2.8%)	
Unclear	334 (19.9%)	589 (59.7%)	
T-sage			< 0.001^b^
T_1_	602 (35.9%)	37 (3.7%)	
T_2_	538 (32.0%)	276 (28.0%)	
T_3_	389 (23.2%)	271 (27.5%)	
T_4_	67 (4.0%)	102 (10.3%)	
Tx	83 (4.9%)	301 (30.5%)	
N-stage			< 0.001^b^
N_0_	1247 (74.3%)	463 (46.9%)	
N_1_	401 (23.9%)	338 (34.3%)	
Nx	31 (1.8%)	186 (18.8%)	
Surgery			< 0.001^b^
Yes	1313 (78.2%)	164 (16.6%)	
No	339 (20.2%)	813 (82.4%)	
Unclear	27 (1.6%)	10 (1.0%)	
Radiation			< 0.001^b^
Yes	52 (3.1%)	74 (7.5%)	
No	1609 (95.8%)	905 (91.7%)	
Unclear	18 (1.1%)	8 (0.8%)	

^a^Chi-square test; ^b^Kruskal-Wallis test

### Metastatic patterns and survival

**Fig. 2:**
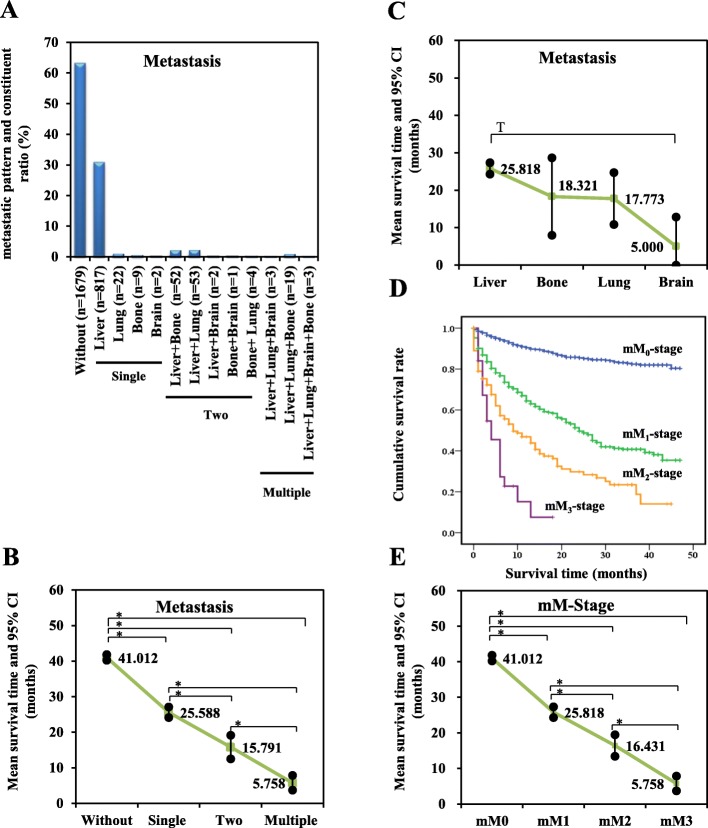
**a** Metastatic patterns of pNENs. **b** Survival time of patients different metastatic patterns. **c** Survival time of patients with single distant organ metastasis. **d** Kaplan-Meier curve of overall survival of patients with modified M-stage classification. **e** Survival time of patients with modified M-stage classification. * Significant difference: *P <* 0.008; ^T^ Tendentious difference: *P* < 0.017

At the time of diagnosis, 1679 (62.98%) patients showed no metastasis. As shown in Fig. 2a, single organ metastases comprised 850 (31.88%) patients, including 817 liver (30.64%), 22 lung (0.83%), nine bone (0.34%), and two brain (0.07%) cases. One hundred and twelve patients (4.20%) showed two-organ metastases, including 52 liver plus bone (1.95%), 53 liver plus lung (1.99%), four bone plus lung (0.15%), two liver plus brain (0.08%), and one bone plus brain (0.04%) cases. Twenty-five patients (0.94%) presented multiple organ metastases, including 19 cases of liver plus lung plus bone (0.71%), three cases of liver plus lung plus brain (0.11%), and three cases of liver plus lung plus brain plus bone (0.11%).

To assess survival time of different metastatic patterns, we compared the survival time of patients without metastasis to those with single distant organ metastasis, two-organ metastases, and multiple organ metastases. As the number of metastatic organs increased, survival time was significantly (*P* < 0.001) reduced (Fig. 2b). In addition, patients with only liver metastasis had a longer survival time than did other single-organ metastases (Fig. 2c), whereas patients with bone, lung or two-organ metastasis had similar mean survival time (bone, 18.32 months ± 5.27 months; lung, 17.77 months ± 3.54 months, two organs metastases, 15.79 months ± 1.70 months).

### Modified M-stage classification and discriminatory capability

Thus, based on the observed metastatic patterns and survival times, we modified the M-stage classification (mM-stage) as shown in Table 2. Tumor without metastasis was defined as mM_0_-stage. Tumor spread from pancreas only to liver was defined as mM_1_-stage. Tumor spreading from pancreas to other single distant organ or to two distant organs was defined as mM_2_-stage. Tumor spreading to three or more distant organs was defined as mM_3_-stage.

**Table 2:** Definition of M-stage classifications

AJCC and ENTES M-stage classifications	Modified M-stage classifications
M_0_-stage, no distant metastasis M_1_-stage, distant metastasis	mM_0_-stage, no distant metastasis mM_1_-stage, only liver metastasis mM_2_-stage, other single distant organ or two organs metastases mM_3_-stage, three or more organs metastases

*AJCC* American Joint Committee on Cancer; *ENETS* European Neuroendocrine Tumor Society

To evaluate survival time among mM-stage classifications, survival curves were plotted using the Kaplan-Meier estimator and then compared with the log-rank test. We observed that all survival curves were well separated (Fig. 2d). Patients with advanced mM stages (mM_1_, mM_2_, mM_3_) had significantly (*P* < 0.001) shorter survival times than patients with mM_0_-stage (Fig. 2e). Moreover, the modified M-stage classification was an independent prognostic factor for pNENs, after adjusting for other clinical and pathological characteristics (Table 3).

**Table 3:** Independent Prognostic Factors

	Univariate		Multivariate	
	HR and 95% CI	*P*-value	HR and 95% CI	*P*-value
Age (years)				
≤ 60	Reference		Reference	
> 60	1.875 (1.595–2.204)	< 0.001	1.744 (1.479–2.055)	< 0.001
Sex				
Male	Reference		Reference	
Female	0.808 (0.691–0.945)	0.008	0.850 (0.726–0.996)	0.044
Race				
White	Reference		Reference	
Black	1.345 (1.082–1.670)	0.007	1.247 (0.998–1.558)	0.052
Other	0.715 (0.527–0.970)	0.031	0.767 (0.565–1.043)	0.090
Size (cm)				
≤ 2	Reference		Reference	
> 2	2.889 (2.241–3.724)	< 0.001	1.322 (1.009–1.731)	0.043
Unclear	6.799(5.110–9.047)	< 0.001	1.547(1.129–2.119)	0.007
Primary Site			a	
Head	Reference			
Body	0.684(0.530–0.884)	0.004		
Tail	0.682(0.556–0.836)	< 0.001		
Other	1.128(0.929–1.368)	0.223		
Differentiation				
Well	Reference		Reference	
Moderately	1.557 (1.095–2.215)	0.014	1.049 (0.735–1.498)	0.791
Poorly	7.414 (5.608–9.803)	< 0.001	3.349 (2.498–4.489)	< 0.001
Undifferentiated	9.494 (6.113–14.743)	< 0.001	3.166 (2.000–5.011)	< 0.001
Unclear	5.136 (4.179–6.311)	< 0.001	1.626 (1.290–2.048)	< 0.001
T-stage			a	
T_1_	Reference			
T_2_	3.434(2.474–4.766)	< 0.001		
T_3_	3.353(2.399–4.688)	< 0.001		
T_4_	7.082(4.867–10.306)	< 0.001		
Tx	9.288(6.696–12.882)	< 0.001		
N-stage				
N_0_	Reference		Reference	
N_1_	1.679 (1.415–1.993)	< 0.001	1.304 (1.092–1.557)	0.003
Nx	3.732 (3.019–4.613)	< 0.001	1.452 (1.152–1.829)	0.002
Surgery				
Yes	Reference		Reference	
No	8.556 (6.941–10.548)	< 0.001	3.901 (3.013–5.050)	< 0.001
Unclear	1.991 (0.812–4.883)	0.133	1.487 (0.601–3.680)	0.391
Radiation			a	
Yes	Reference			
No	1.984(1.511–2.603)	< 0.001		
Unclear	0.939(0.420–2.098)	0.878		
mM-stage				
mM_0_-stage	Reference		Reference	
mM_1_-stage	4.520 (3.789–5.393)	< 0.001	1.643 (1.339–2.016)	< 0.001
mM_2_-stage	8.199(6.380–10.537)	< 0.001	2.249(1.704–2.968)	< 0.001
mM_3_-stage	16.356 (10.266–26.059)	< 0.001	5.034 (3.110–8.150)	< 0.001

^a^variables excluded by multivariate forward stepwise cox regression

To explore discriminatory capability of the modified M-stage classification, Harrell’s concordance index was calculated. The mM-stage classification had a better discriminatory capability (Harrell’s concordance index, 0.712; 95% CI, 0.692–0.732) than AJCC M-stage and ENETS M-stage (Harrell’s concordance index, 0.697; 95% CI, 0.678–0.717).

## Discussion

In agreement with previous studies [9–11], the present study also demonstrated that nearly one quarter of patients (37.02%, 987/2666) presenting metastasis at the time of pNEN diagnosis. In addition, liver metastasis was the majority metastatic pattern, followed by lung, bone and brain metastasis. The hematogenous mode of metastasis might contribute to the metastatic pattern, which we have observed in the present study. Unsually, carcinoma cells seed in the liver via the portal venous system. Then, these cells would spread to lung via the inferior vena cava and pulmonary arteries. Finally, the carcinoma cells from lung metastases would seed in other organs via arterial blood [12].

The present study found that with an increasing number of metastatic organs, there was a significant decrease in survival time. In addition, pNENs with liver metastasis had longer overall survival than other single-organ metastatic patterns. However, AJCC and ENETS classify both pNENs with liver metastasis and pNENs with the other metastasitic patterns as M_1_-stage. Our modified M-stage classification distinguishes that tumor spreading from pancreas only to liver should be separated from the other metastatic patterns, and that it is necessary to design individualized treatment and follow-up programs for patients with lung, bone, or brain metastasis.

Usually, pancreatic resection is not performed when the pancreatic malignant tumor has spread to other organs [13]. However, considering the indolent behavior of pNENs and the high frequency of liver metastasis, several clinicians suggested surgical management could give rise to benefit to pNENs with liver metastasis [4, 14]. Birnbaum et al. pancreatic resection could slow down tumor growth and reduce hormone production [14], possibly resulting in considerable benefit for patients with liver metastasis [4].

Consistent with previous studies, the tumor size, primary site, differentiation, AJCC T-stage and AJCC N-stage were identified as predictors of distant organ metastasis (Additional file 1: Table S1). Unfortunately, SEER database did not record Ki-67 status and graded the primary tumor only on the basis of morphological description (ICD-O-3) in the pathology report. Thus, we failed to evaluate the predictive role of Ki-67 status and WHO 2010 grading classification (NET G1, NET G2, NET G3 and NEC) in distant organ metastasis.

It seems the primary tumor site is a particularly useful predictor because it is available before any operation occurs. Hao et al. reported that compared to tumors located in the head and neck of the pancreas, tumors in the body and tail showed a decreased risk of liver metastasis in pancreatic adenocarcinoma [15]. In contrast, the present study showed that pNENs located in the pancreatic tail are actually 1.728 times more likely (*P* < 0.001) to develop metastasis, as compared to tumors located in the pancreatic head. This may be due to the fact that patients with pNENs, especially non-functioning pNENs, in the tail of the pancreas are less likely to experience obstructive signs and hormonal symptoms until tumors spread to the peritoneum, spleen, and distant organs [16, 17]. Thus, at the time of diagnosis, distant organ metastases exist in most of these patients.

Some limitations of the present study should be noted. First, the SEER database only provides information on pNEN metastasis to liver, lung, bone, and brain. The frequency of pNEN metastasis might be underestimated. Second, Hlatky et al. noted that multiple metastatic lesions may be related to a short survival time [18]. However, the SEER database did not collect data on the number of metastatic lesions in each distant organ.

## Conclusions

In conclusion, this is the first population-based study to investigate the metastatic patterns and predictors in advanced pNENs. We found significant differences in survival time across different metastatic patterns. Thus, the modified M-stage classification show a better discriminatory capability than the AJCC and ENETS M-stage classifications. In the future, clinicians should determine individualized treatment and follow-up programs for pNENs with different metastatic patterns.

## Additional file

Additional file 1: **Table S1.** Clinicopathological characters associated with metastasis. (DOCX 22 kb)

## Abbreviations

AJCC: American Joint Committee on Cancer
ENETS: European Neuroendocrine Tumor Society
OS: Overall survival
pNENs: Pancreatic neuroendocrine neoplasms
SD: Standard deviation
SEER: Surveillance, Epidemiology, and End Result
