# Supplementary material for: A modified M-stage classification based on the metastatic patterns of pancreatic neuroendocrine neoplasms: a population-based study
Source: BMC Endocr Disord. 2018 Oct 19;18:73. doi: 10.1186/s12902-018-0301-z (PMC6194708; doi:10.1186/s12902-018-0301-z)
Supplement: Supplementary file 1 — Table S1. Clinicopathological characters associated with metastasis. (DOCX 22 kb) [file 12902_2018_301_MOESM1_ESM.docx]

**Additional file 1: Table S1**

| ` | Univariate | | Multivariate | |
| --- | --- | --- | --- | --- |
|  | **OR and 95% CI** | ***P*-value** | **OR and 95% CI** | ***P*-value** |
| Age (years) |  |  |  |  |
| ≤ 60 | Reference | | † | |
| > 60 | 1.104 (0.942-1.292) | 0.221 |  |  |
| Sex |  |  |  |  |
| Male | Reference | | † | |
| Female | 0.926 (0.790-1.084) | 0.338 |  |  |
| Race |  | |  | |
| White | Reference | | † | |
| Black | 1.136 (0.894-1.444) | 0.296 |  |  |
| Other | 0.672 (0.502-0.899) | 0.007 |  |  |
| Size (cm) |  | |  | |
| ≤ 2 | Reference | | Reference | |
| > 2 | 7.374 (5.640-9.640) | < 0.001 | 3.154 (1.808-5.501) | < 0.001 |
| Unclear | 28.902(20.128-41.501) | < 0.001 | 1.916(1.041-3.527) | 0.037 |
| Primary Site |  | |  | |
| Head | Reference | | Reference | |
| Body | 0.699(0.535-0.914) | 0.009 | 1.046(0.751-1.457) | 0.790 |
| Tail | 1.127(0.919-1.383) | 0.251 | 1.728(1.343-2.225) | < 0.001 |
| Other | 1.768(1.426-2.193) | < 0.001 | 1.459(1.107-1.922) | 0.007 |
| Differentiation |  | |  | |
| Well | Reference | | Reference | |
| Moderately | 2.147 (1.601-2.880) | < 0.001 | 1.696 (1.235-2.328) | 0.001 |
| Poorly | 8.192 (5.756-11.657) | < 0.001 | 4.853(3.309-7.117) | < 0.001 |
| Undifferentiated | 9.089 (4.879-16.934) | < 0.001 | 5.757 (2.935-11.292) | < 0.001 |
| Unclear | 9.732 (7.928-11.946) | < 0.001 | 6.251 (4.944-7.904) | < 0.001 |
| T-stage |  | |  | |
| T_1_ | Reference | | Reference | |
| T_2_ | 8.347(5.810-11.991) | < 0.001 | 1.802(0.920-3.530) | 0.086 |
| T_3_ | 11.335(7.858-16.351) | < 0.001 | 2.747(1.431-5.274) | 0.002 |
| T_4_ | 24.770(15.747-38.963) | < 0.001 | 3.700(1.803-7.596) | < 0.001 |
| Tx | 59.004(39.103-89.033) | < 0.001 | 8.776(4.529-17.007) | < 0.001 |
| N-stage |  |  |  |  |
| N_0_ | Reference | | Reference | |
| N_1_ | 2.270 (1.897-2.717) | < 0.001 | 1.527 (1.223-1.908) | < 0.001 |
| Nx | 16.160 (10.888-23.985) | < 0.001 | 4.836 (3.047-7.676) | < 0.001 |

† variables excluded by multivariate forward stepwise cox regression
